# Editorial: Novel antimicrobials and antibiotics resistance modulating agents from natural products: Turning promises into Triumphs

**DOI:** 10.3389/fphar.2023.1184071

**Published:** 2023-04-11

**Authors:** Nada M. Mostafa, Muhammad Ayaz, Mohamed El-Shazly, Abdel Nasser B. Singab

**Affiliations:** ^1^ Department of Pharmacognosy, Faculty of Pharmacy, Ain Shams University, Cairo, Egypt; ^2^ Department of Pharmacy, Faculty of Biological Sciences, University of Malakand, Chakdara, Pakistan; ^3^ Center of Drug Discovery, Research and Development, Faculty of Pharmacy, Ain Shams University, Cairo, Egypt

**Keywords:** natural products, antimicrobials, antibiotic resistance, anti-biofilm agents, computational studies, nanotechnology, biotechnology

With the global rise of antibiotics-resistant microbes and the ability of many microbial strains to form complex biofilms, researchers have been encouraged to search for natural products with reported antibiofilm capabilities as safer alternatives to hazardous chemicals and good resources for new antimicrobial leads (Edmond et al., 2021). Natural antimicrobials can be life-saving if well-implemented, literature suggested their efficacy in tackling food-borne pathogens, fungal infections, urinary tract diseases, and many others (El-Nashar et al., 2021a; El-Nashar et al., 2021b; Edmond et al., 2021). They can also target infected organs by loading on different carriers *via* implementation of nanotechnologies providing higher bioavailability, efficient delivery and reducing drug doses (Zhang et al., 2021). We cannot also ignore the increasing applications of biotechnology in this regard, including the modulation of natural products on molecular basis and the production of antimicrobials from bacteria and fungi rendering this approach a promising arena in modern therapy (Ahmed Sheikh, 2010). Natural products can be obtained from different sources as plants, animals, marine organisms and their associated endophytic bacteria and fungi (Ayoub et al., 2010; Mostafa et al., 2021). They contain a variety of secondary metabolites with interesting bioactivities (Mostafa, 2018; Moussa et al., 2020) and are thus under consideration for the discovery and development of novel drugs against various disorders including infectious diseases (Ayaz et al., 2017; Ovais et al., 2019b; Mostafa et al., 2022).

An impressive number of research articles and review manuscripts were submitted to this Research Topic for consideration and a total of seven manuscripts were accepted for publication. Mohsin et al. ) analyzed the anti-onychomycosis potential of *Allium sativum, Zingiber officinale, Nigella sativa, Curcuma longa, Withania somnifera, Azadirachta indica,* and *Lawsonia inermis.* Crude samples were phytochemically analyzed *via* RP-HPLC revealing rutin, gallic acid, catechin, syringic acid, emodin, luteolin, myricetin, quercetin, and antifungal potential against dermatophytes, non-dermatophytes, *Candida albicans,* and yeasts was evaluated. Furthermore, the synergistic efficacy of amphotericin-B and terbinafine was checked against different fungi *via* time-kill kinetics and proteins estimation. Tested samples showed anti-radical and antifungal potential against both dermatophytes and non-dermatophytes. Synergistic effect was observed between extracts and standard antifungal drugs with a 4-8-fold decline in the MICs, revealing time and dose-dependent antifungal effect. The inhibition of fungal protein was more dominant in synergy-group compared to extracts-groups alone.

Medicinal plants are rich in metabolites that exhibit diverse antimicrobial potentials (Shah et al., 2019; Zohra et al., 2019). Abdelgawad et al. reported the potential efficacy of olive leaves and their phytochemicals against SARS-CoV-2. Olive leaves contain phytoconstituents as oleuropein, apigenin-7-O-glucoside, verbascoside, hydroxytyrosol, and luteolin-7-O-glucoside and triterpenoids like ursolic acid, oleanolic acid, and maslinic acid which have known anti-viral efficacy. They are also reported to exhibit anti-inflammatory properties which strengthen their potential against SARS CoV-2 mediated coagulatory infections and associated co-morbidities.

Li et al. tested the efficacy of 126 coumarin derivatives as potential anti-viral agents against Hantaan virus (HTNV) and their mechanism. Among the tested compounds, N6 exhibited considerable anti-HTNV activity with high selectivity index (10.9) and a decline in the viral titer at 5, 10, and 20 μM. N6 administration during early infection stages inhibited viral replication in the host cells as indicated by the results from the HTNV-infected newborn mice. Molecular docking studies revealed that N6 interacted with high affinity to the amino acids’ active sites. Treatment with N6 was observed to be mediated *via* inhibition of p (Ser473)Akt and HTNV nucleocapsid proteins expression.

Li et al. performed high-throughput screening of the natural products library against a tick-borne fatal viral infection called severe fever with thrombocytopenia syndrome virus (SFTCV). The study identified three compounds (notoginsenoside Ft1, toosendanin, and punicalin) with high efficacy against SFTV. Toosendanin was the most potent; it interfered with the viral replication at internalization stage. Toosendanin also reduced the *in-vivo* viral load inside the infected animals with considerable histopathological changes. Moreover, it was subjected to anti-viral studies against bunyavirus and SARS-CoV-2 infections and a broad anti-viral effect was observed, thus signifying further studies for its potential clinical use.

Dahibhate et al. reported the anti-*Pseudomonas aeruginosa* potential of cyclic disulfide diastereomer isolated from *Bruguiera gymnorhiza* bioactive fraction (BG138). BG138 was phytochemically characterization by GC-MS, IR, ^1^H-NMR, and ^13^C-NMR revealing the presence of brugierol and isobrugierol. BG138 was tested against microbial virulence factors, biofilm formation, expression of QS-related genes and quorum sensing molecules. BG138 exhibited antibacterial (MIC of 32 μg/mL) and dose-dependent anti-biofilm activities with bacterial morphological changes and improved propidium iodide uptake by the bacterial cells. Furthermore, it inhibited bacterial virulence factors with reduced motility and swimming potential. The expression of anti-quorum sensing genes and cell damaging potential against the bacteria revealed it as a potential source of novel bioactive resistance modifying compounds.

Green nanotechnology is an emerging field of research to improve target drug delivery and bioavailability (Ovais et al., 2019a; Khalil et al., 2021; Patra et al., 2021). Djearamane et al. studied the efficacy of zinc oxide nanoparticles (ZnO-NPs) on bacterial cell walls and their morphological changes using FTIR and SEM techniques. ZnO-NPs cellular accumulation was assessed *via* dispersive X-ray. They exhibited concentration-dependent inhibition of bacterial growth. ZnO-NPs were more effective against *Enterococcus faecalis* than *Serratia marcescens* with percent growth inhibition of 63.50% and 51.27%, respectively. This was associated with bacterial morphological changes, loss of integrity and cell death. In another study, Abbas et al. evaluated the synergistic effect of a phenolic compound propyl gallate in combination with orbifloxacin against the KVCC1423-resistant *Escherichia coli* strain, resulting in reduction of the MIC of orbifloxacin in combination therapy with a 74% decline in MIC of both drugs, considerable inhibition of biofilm formation and bacterial motility. Thus, this combination was observed as a useful synergistic antimicrobial against resistant *E. coli* strains.

In conclusion, this Research Topic’s objective was to consider both research and review papers related to the potential drug discovery of antimicrobial agents from natural sources. This Research Topic offered a new venue for researchers to share their cutting-edge research findings in the field of natural products, novel antimicrobial agents and antibiotic resistance modulators.

## References

[B1] Ahmed Sheikh H. M. (2010). Antimicrobial activity of certain bacteria and fungi isolated from soil mixed with human saliva against pathogenic microbes causing dermatological diseases. Saudi J. Biol. Sci. 17 (4), 331–339. 10.1016/j.sjbs.2010.06.003 30323712 PMC6181150

[B2] Ayaz M. Subhan F. Sadiq A. Ullah F. Ahmed J. Sewell R. D. E. (2017). Cellular efflux transporters and the potential role of natural products in combating efflux mediated drug resistance. Front. Biosci. 22 (4), 732–756. 10.2741/4513 27814643

[B3] Ayoub N. Singab A. N. Mostafa N. Schultze W. (2010). Volatile constituents of leaves of Ficus carica Linn. grown in Egypt. J. Essent. Oil Bear. Plants 13 (3), 316–321. 10.1080/0972060x.2010.10643827

[B4] Edmond M. P. Mostafa N. M. El-Shazly M. Singab A. N. B. (2021). Two clerodane diterpenes isolated from *Polyalthia longifolia* leaves: Comparative structural features, anti-histaminic and anti-*Helicobacter pylori* activities. Nat. Prod. Res. 35 (23), 5282–5286. 10.1080/14786419.2020.1753048 32363939

[B5] El-Nashar H. A. Mostafa N. M. El-Badry M. A. Eldahshan O. A. Singab A. N. B. (2021a). Chemical composition, antimicrobial and cytotoxic activities of essential oils from *Schinus polygamus* (Cav.) cabrera leaf and bark grown in Egypt. Nat. Prod. Res. 35 (23), 5369–5372. 10.1080/14786419.2020.1765343 32441134

[B6] El-Nashar H. A. Mostafa N. M. El-Shazly M. Eldahshan O. A. (2021b). The role of plant-derived compounds in managing diabetes mellitus: A review of literature from 2014 to 2019. Curr. Med. Chem. 28 (23), 4694–4730. 10.2174/0929867328999201123194510 33231145

[B8] Khalil A. T. Iqbal J. Shah A. Haque M. Z. Khan I. Ayaz M. (2021). The bio–nano interface as an emerging trend in assembling multi-functional metal nanoparticles. Spr. Nanosci. 7, 1–24. 10.1039/9781839163791-00001

[B9] Mostafa N. M. (2018). β-Amyrin rich *Bombax ceiba* leaf extract with potential neuroprotective activity against scopolamine-induced memory impairment in rats. Rec. Nat. Prod. 12 (5), 480–492. 10.25135/rnp.47.17.10.062

[B10] Mostafa N. M. Edmond M. P. El-Shazly M. Fahmy H. A. Sherif N. H. Singab A. N. B. (2022). Phytoconstituents and renoprotective effect of *Polyalthia longifolia* leaves extract on radiation-induced nephritis in rats via TGF-β/smad pathway. Nat. Prod. Res. 36 (16), 4187–4192. 10.1080/14786419.2021.1961252 34491152

[B11] Mostafa N. M. Mostafa A. M. Ashour M. L. Elhady S. S. (2021). Neuroprotective effects of black pepper cold-pressed oil on scopolamine-induced oxidative stress and memory impairment in rats. Antioxidants 10 (12), 1993. 10.3390/antiox10121993 34943096 PMC8698347

[B12] Moussa A. Y. Mostafa N. M. Singab A. N. B. (2020). Pulchranin A: First report of isolation from an endophytic fungus and its inhibitory activity on cyclin dependent kinases. Nat. Prod. Res. 34 (19), 2715–2722. 10.1080/14786419.2019.1585846 30887847

[B13] Ovais M. Khalil A. T. Ayaz M. Ahmad I. (2019a). “Biosynthesized metallic nanoparticles as emerging cancer theranostics agents,” in Nanotheranostics: Applications and limitations (Berlin: Springer Nature), 229–244.

[B14] Ovais M. Zia N. Khalil A. T. Ayaz M. Ahmad I. Khalil A. B. (2019b). “Nanoantibiotics: Recent developments and future prospects,” in Frontiers in clinical drug research - anti-infectives (Netherlands: Bentham Science Publishers), 30–54.

[B15] Patra C. Ahmad I. Ayaz M. Khalil A. T. Mukherjee S. Ovais M. (2021). Biogenic nanoparticles for cancer theranostics. Amsterdam: Elsevier.

[B16] Shah S. M. Ullah F. Ayaz M. Wahab A. Shinwari Z. K. (2019). Phytochemical profiling and pharmacological evaluation of Ifloga spicata (forssk.) Sch. Bip. In leishmaniasis, lungs cancer and oxidative stress. Pak. J. Bot. 51 (6), 2143–2152. 10.30848/pjb2019-6(39)

[B17] Zhang R. Liu F. Tian Y. Cao W. Wang R. (2021). Editorial: Nanotechnology in traditional medicines and natural products. Front. Chem. 9, 633419. 10.3389/fchem.2021.633419 33692985 PMC7937879

[B18] Zohra T. Ovais M. Khalil A. T. Qasim M. Ayaz M. Shinwari Z. K. (2019). Extraction optimization, total phenolic, flavonoid contents, HPLC-DAD analysis and diverse pharmacological evaluations of Dysphania ambrosioides (L.) Mosyakin & Clemants. Nat. Prod. Res. 33 (1), 136–142. 10.1080/14786419.2018.1437428 29430965

